# Local and scientific knowledge in the school context: characterization and content of published works

**DOI:** 10.1186/s13002-020-00373-5

**Published:** 2020-05-06

**Authors:** Maria Carolina Sotero, Ângelo Giuseppe Chaves Alves, Janaina Kelli Gomes Arandas, Maria Franco Trindade Medeiros

**Affiliations:** 1 Programa de Pós-Graduação em Etnobiologia e Conservação da Natureza, Universidade Federal Rural de Pernambuco, Rua Dom Manoel de Medeiros, s/n, Recife, PE 52171-900 Brazil; 2 Departamento de Ecologia, Universidade Federal Rural de Pernambuco, Rua Dom Manoel de Medeiros, s/n, Recife, PE 52171-900 Brazil; 3 Departamento de Zootecnia, Universidade Federal Rural de Pernambuco, Rua Dom Manoel de Medeiros, s/n, Recife, PE 52171-900 Brazil; 4 Departamento de Botânica, Museu Nacional da Universidade Federal do Rio de Janeiro, Quinta da Boa Vista, s/n 20.940-040, Rio de Janeiro, RJ Brazil

**Keywords:** Database searches, Scientometrics, Ethnobiology, Teaching-learning, Contextualized education, Multiculturalism

## Abstract

**Background:**

Bridging the gap between local and scientific knowledge can have useful implications in the teaching-learning process because it can create environments conducive to the valorization of sociocultural diversity in schools. The present review aims to analyze the profile and contributions of scientific publications dealing with articulations between local and scientific knowledge in basic formal education.

**Method:**

Combined searches of 14 terms related to ethnoscience and 20 terms of education were conducted in English, Portuguese, and Spanish using the databases of *Web of Science*, *Scopus*, *Science Direct*, and *Scielo*. The recovered works were filtered, organized in a spreadsheet, and analyzed for publication characteristics (year, author, periodicals, countries of origin of the authors, and countries surveyed) and contents of the studies (epistemological bases, techniques of application, and record of the articulation of local and scientific knowledge).

**Results:**

The research field that establishes these articulations is growing, with 81% of the works being written in the English language. A total of 494 researchers were recorded. The USA, South Africa, Brazil, Canada, and Australia were the countries of origin of the first author for the majority (64%) of the works considered. Multiculturalism, Vygotskian theory of learning, postcolonial theory, constructivism, critical pedagogy, and the argumentation theory were the main theoretical bases of half of the recovered works in which some explicit theoretical orientation could be found. Teacher training and interviews stood out as important tools in the application and record of links between local and scientific knowledge, respectively.

**Conclusions:**

Interdisciplinary approaches were common in the conception and application of pedagogical activities reported in the recovered works. Articulations between local and scientific knowledge are effective for culturally-sensitive scientific education, especially (but not exclusively) in schools directly related to traditional communities. There was a tendency to emphasize the teacher as a fundamental agent in the search for education that establishes these articulations. The authors of the analyzed works frequently indicated a need for greater proximity of the community to school spaces.

## Background

Each human society, while dealing with natural resources in regular daily life, creates a unique body of knowledge [1]. Such knowledge may be referred to as indigenous, tribal, traditional, native, or rural, among others [2]. Here, we use the term “local knowledge.” This term refers to knowledge that is based on experience and reproduced in a culturally specific environment [3]. Therefore, this knowledge is different from scientific knowledge, which is developed through controlled experimentation and is produced within formal institutions [4].

In schools, aspects of local knowledge can be found in previous student knowledge [5], in multicultural curricula, and in the everyday practices of local communities. Schools, therefore, are spaces where students and teachers have the opportunity to realize in practice how science and other forms of knowledge may connect and benefit each other [6].

A concrete example can be found in an experience in Northeast Brazil, as reported by Baptista [7]. Using interviews, the author accessed previous knowledge brought to school by students who were also local farmers. From this, a didactic tool was developed which was used to compare scientific and local names of plant structures and parts. The tool was also used to discuss physiological and morphological changes in plants that the students observed in their everyday farming experience [7].

Another example is an experience with the Adi people in India, in which students were encouraged to interview local elderly people about plants that could be used as food. A recipe contest was then held using those plants as a reference. Thus, in this way, cultural information on the use of food plants was recovered and organized. Scientific learning was also fostered through the establishment of a herbarium [8].

However, studies have indicated the existence of a gap between school life, based only on scientific content, and the daily life of students, supported by local knowledge, which are not always called into dialogue [9, 10]. Situations of asymmetrical articulation are sometimes reported, where the inclusion of local knowledge in the teaching-learning process is carried out so as to “fit” into science, under the conditions of respect for established limits and perpetuation of the authority of scientific knowledge [11].

Schooling has been analyzed in two distinct ways in relation to local knowledge: sometimes as one of the causes of its erosion, as it opens a new generation to other forms of seeing the world; and as a possible solution against its disappearance, under the condition of a curriculum that values the local culture and its peculiarities [12].

The inclusion of local knowledge in the teaching-learning process can facilitate the understanding of subjects being developed on the conceptions of science, which are often distant from student experiences, and thus can represent a first step to opening doors to scientific literacy [13]. In this way, local knowledge constitutes a pedagogical, instructional, and communicative tool for the educator [14].

Articulating local and scientific knowledge leads students to a broader view of the world [15], and encourages respect for socially constructed forms of thought. Students are sociocultural subjects that, when included in the school environment, bring with them knowledge, cultures, and more-or-less conscious projects, as a result of their experiences [16].

Given the pertinence of establishing relationships among different types of knowledge in the teaching-learning process in the school context, a systematic analysis of the characteristics of publications with this as a theme would be beneficial. Such an analysis would contribute to a better understanding of the advances in this field, as well as indicate trends or possible new directions. It would also contribute to the construction of contextualized and culturally sensitive education.

The aim of the present study was to identify and characterize studies that made articulations between local and scientific knowledge in the school context in basic formal education. Furthermore, this study aimed to analyze the different contributions to the teaching-learning process that emerged from this relationship in the recovered works.

Thus, the following questions were considered: What are the general features of the set of recovered works? What are the arguments most frequently used by authors about the relevance of articulations between types of knowledge in formal education? What are the theoretical and methodological supports used by the authors in their approach to these articulations?

## Methodology

In order to obtain a general characterization of what has already been published regarding the scope of our objectives, the following aspects were considered: total number, language and distribution of published works over time (years) and space (countries), and vehicles used for publication (journals, books, and proceedings) and frequency of certain selected search terms.

Search terms were selected by consulting publications that made the connection between local and scientific knowledge in the school context in the field of ethnoscience. Some terms were also gathered from previous work by Baptista and El-Hani [7], El-Hani and Mortimer [17], and El-Hani and Bizzo [18], because we recognize Charbel El-Hani as one of the pioneers in the study of ethnoscience and education, and who still stands out in the field till today.

The selection of terms and the accomplishment of pilot research followed the same methodological sequence carried out by Bartol and Mackiewicz-Talarczyk [19].

Thus, this first phase resulted in the selection of 14 terms from the field of ethnoscience and 20 from the field of education that could be efficient in the search for studies that related local and scientific knowledge in the school context (see Table 1).

**Table 1:** Terms related to ethnoscience and education selected in the first phase

Terms related to ethnoscience	Terms related to education
Biocultural evolution Biocultural knowledge Cultural transmission Ecological knowledge Ethnobiology Ethnobotany Ethnoecology Ethnopedology Ethnozoology Indigenous knowledge Local knowledge Traditional ecological knowledge Traditional knowledge Native knowledge	Aboriginal education Aboriginal school Alternative conception Conceptual profile Contextualized education Education Epistemological pluralism Indigenous education Indigenous school Multiculturalism Pedagogical practice Rural education School School knowledge Science classes Scientific education Student Teacher Teaching learning Urban education

While searching the word “education” combined with terms related to the ethnosciences, the word “school” was added. So, in that case, the final search was carried out combining ethnoscience-related terms, on one side, with the words “education” and “school” on the other side. This was done in order to maintain the focus of the study, since the objective was related to the school context.

In the second phase of the review, each term we had included in the first column of Table 1 was combined with each of the terms in the second column for searches of the following databases: *Web of Science* (www.webofknowledge.com), *Scopus* (www.scopus.com), *Science Direct* (www.sciencedirect.com), and *Scielo* (www.scielo.org).

To increase search specificity, the combined terms were enclosed in quotation marks, and their variations were made plural when applicable. Since combined searches were employed, each term in column 1 was searched together with each term of column 2, using the Boolean operator “AND”, as in the research by Barreto et al. [20].

The four databases included indexed journals in ten different areas: Agrarian Sciences, Biological Sciences, Health Sciences, Exact and Earth Sciences, Human Sciences, Applied Social Sciences, Engineering, Linguistics, Letters and Arts, and Multidisciplinary [21].

Searches were carried out in English, Portuguese, and Spanish in each of the databases. The pertinence of including the latter two languages is the large number of publications in the field of ethnoscience carried out by researchers whose origins are from countries such as Brazil, Argentina, and Mexico [22], besides the fact that Spanish, along with English, is one of the languages of global communication.

Thus, a total of 3360 searches of studies were carried out (14 ethnoscience terms combined with 20 education terms in three languages using four databases). Searches were carried out in January 2018 until December 2019.

All publications were considered from the beginning of coverage by each database until publications of December 2017. No filters were applied regarding the areas of knowledge within the databases and all types of publications that the bases covered were considered, that is, articles of scientific journals, book chapters, and conference proceedings, which were treated equally in our study as “works.”

The works resulting from the searches were first filtered by titles and abstracts, followed by a subsequent screening based on analysis of the complete contents of the works. The works were ultimately organized in electronic spreadsheets.

Only works that somehow related local knowledge with scientific knowledge in the basic school (elementary and high school) context were included, while works directed toward university education, such as that of Mulej and Sirca (2010) [17], were not. This decision was justified by differences in relation to basic education regarding the characteristics of the agents involved (teachers and students), public policies, and curricula. This decision was only methodological and does not diminish the scientific relevance of such publications, nor the perspective of them being used as a pedagogical tool, continuous training material for teaching staff, or a theoretical tool for the development of public education policies.

It should be noted that, for methodological purposes, works on the perception or transmission of knowledge that used the school environment as a place of study, but whose objectives and results were not directly linked to the teaching-learning perspective, such as Pontes-da-Silva [23] for example, were not included in this research.

The frequencies of works/year were submitted to regression analysis to obtain an equation for data prediction and the evaluation of changes over time. The dependent variable was the frequency of works that established articulations between local and scientific knowledge in the school context, while the independent variable was year of publication. We opted for the polynomial model, since it was the one that best fit the estimation of this information according to the coefficient of determination (*R*^2^). The journals in which the works had been published were characterized by their impact factor and h-index, which was obtained from Google Scholar (http://scholar.google.com) and Scimago Journal and Country Rank (www.scimagojr.com/journalsearch). The impact factor corresponds to the average number of times the article in question has been cited in the last 2 years [24]. The h-index (h5) analyzes all articles published in a given periodical in a given period of time, compared with the total citations of the articles contained therein [25].

The country of origin of the first author was identified from the corresponding address provided in the works and/or searches with the full name of the researcher in general and specific search sites.

Multivariate analysis of simple correspondence was performed to determine associations between the frequencies of countries of origin of first authors and the frequencies of countries in which the surveys were carried out. The option to consider the first author followed the same methodology as Campos et al. [22]. Only five countries of origin were thus considered: the USA, South Africa, Brazil, Canada, and Australia. These countries were selected because they were the place of origin of more than 64% of the authors of the recovered works. The software TIBCO Statistica, version 13.3, was used in this analysis.

Correspondence analysis is usually used to associate all categories of a variable with all categories of another variable, generating a graphical representation in which closely located categories have stronger relationships than those that are more distant [26].

In order to quantify the presence of terms in the content of works, the simple frequency of works recovered from combinations of terms using the English language was determined.

Finally, the considered works were analyzed to characterize the content of the studies in question, raising information on some aspects such as the critical incorporation of local knowledge in the formal teaching process; the results obtained in order to articulate local and scientific knowledge in the school context; suggestions for future work; the epistemological visions that guided the work; and the methodological techniques used.

The methodological techniques adopted by the authors of the works were separated into two groups: those used by the researchers during activities to establish integrations between local and scientific knowledge (treated in this study as application techniques) and those performed later by the researchers to understand the results generated in these activities (here called evaluation techniques).

## Results

### General characterization of works

Grouping the works recovered from the four databases, and excluding those that were repeated, resulted in a final total of 266 studies that related local and scientific knowledge in the school context. Works were mostly written in English (81.2%), followed by Portuguese (9.8%) and Spanish (8.3%). Two works were recovered in French (0.8%) and one in Afrikaans (0.4%), which occurred by using the English language in their abstracts and keywords.

**Fig. 1:**
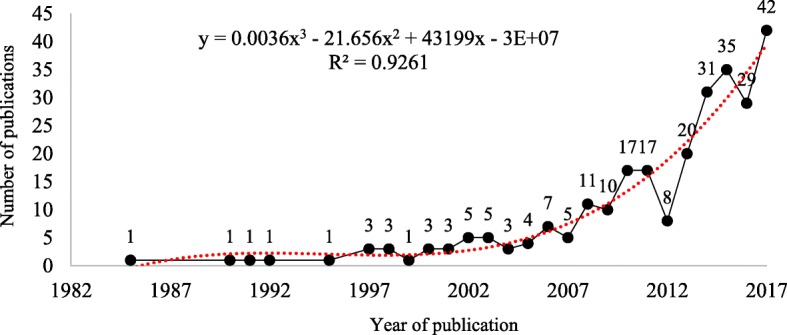
Number of works that establish articulations between local and scientific knowledge (1985 to 2017). Source: Database searches of *Web of Science*, *Scopus*, *Science Direct*, and *Scielo*

The earliest work recovered was the article titled “*Science*, *health and everyday knowledge*: *a case study about the common cold*,” published in the *European Journal of Science Education* in 1985 by sociologist Alan Prout. During the subsequent 22 years (from 1985 to 2007), the annual frequency of recovered works did not exceed seven per year. This was followed by a general increasing trend from 2008 to 2017. The polynomial model provided the best fit (*R*^2^ = 0.92) (see Fig. 1).

Analysis of means of dissemination revealed that 93% of the recovered studies were published in journals (*n* = 247), followed by 4% in books (*n* = 12) and 3% in conference proceedings (*n* = 8). The published books were in the social sciences area, while conferences were also in this area as well as in education and technology.

The works published in journals were distributed among 159 different vehicles, of which ten journals had more than three related articles. The word “education” was present in the title of eight of these ten journals (see Table 3).

The journals with the most studies that related local and scientific knowledge in the school context were *Cultural Studies of Science Education* (16 articles), followed by *African Journal of Research in Mathematics*, *Science and Technology Education* (ten articles), and *International Journal of Science Education* (nine articles) (see Table 2).

**Table 2:** Frequency of works that related local and scientific knowledge and scientometric indices for journals

#	Scientific journals (*n* = 159^a^)	FREQ (*n*)	%	Impact factor	H-Index
1	Cultural Studies of Science Education	16	6.5	0.931	23
2	African Journal of Research in Mathematics, Science and Technology Education	10	4.0	0.545	12
3	International Journal of Science Education	09	3.6	1.611	93
4	Ciência & Educação	07	2.8	b	17
5	Procedia - Social and Behavioral Sciences	06	2.4	b	39
6	International Journal of Science and Mathematics Education	05	2.0	1.399	31
7	Journal of Ethnobiology and Ethnomedicine	05	2.0	2.504	57
8	Journal of Geoscience Education	04	1.6	1.014	28
9	Science Education	04	1.6	2.897	100
10	The Australian Journal of Indigenous Education	04	1.6	0.938	18

*FREQ* total number of occurrences of related works in the journal. Impact factor = metric that assesses the impact of academic journals based on the citation counts created [27]. H-Index = analyzes all articles published in a given journal in a given period of time, compared to the total citations of the articles contained therein [25]

^a^Only journals that had more than three publications related to the present study are listed

^b^Data not found in *Scimago Journal and Country Rank* and/or in *Google Scholar*.

Source: Databases *Web of Science*, S*copus*, *Science Direct* and *Scielo*, and on-line platforms *Scimago Journal and Country Rank* [27] and *Google Scholar* [28]

The high frequency of articles published in the journals *Cultural Studies of Science Education* and *African Journal of Research in Mathematics*, *Science and Technology Education* reveals their importance as vehicles of information on the connections among different types of knowledge in the school environment (see Table 2).

A total of 494 researchers, both authors and collaborators who worked on the connection between local and scientific knowledge in the school context, were recorded. The authors who have been dedicating themselves to this theme, who have published more related works, and who can serve as key authors for the study and understanding of research with this theme are Meshach Bolaji Ogunniyi of University of the Western Cape (South Africa) and Victoria Reyes-García of Universitat Autônoma de Barcelona (Spain) (six and five, respectively) followed by Glen S. Aikenhead of University of Saskatchewan (Canada) and Geilsa Costa Santos Baptista of Universidade Estadual de Feira de Santana (Brazil) (four works each).

The first authors of the recovered works were from 42 countries while their research was conducted in 48 countries (plus the Arctic region, where research did not clearly describe the entire territory covered). Study areas were not presented in 63 works because they were literature reviews.

Excluding review works, the countries of the first authors were, in decreasing order, the USA (*n* = 44), South Africa (*n* = 31), Brazil (*n* = 27), Canada (*n* = 18), and Australia (*n* = 11), which together accounted for 64% of the works that established articulations between local and scientific knowledge in the school context.

Brazil, South Africa, and Australia were the countries in which researchers were most likely to perform research in their own territory (89%, 87%, and 73%, respectively), while in the USA and Canada this phenomenon occurred in 61% and 44% of recovered works, respectively.

**Fig. 2:**
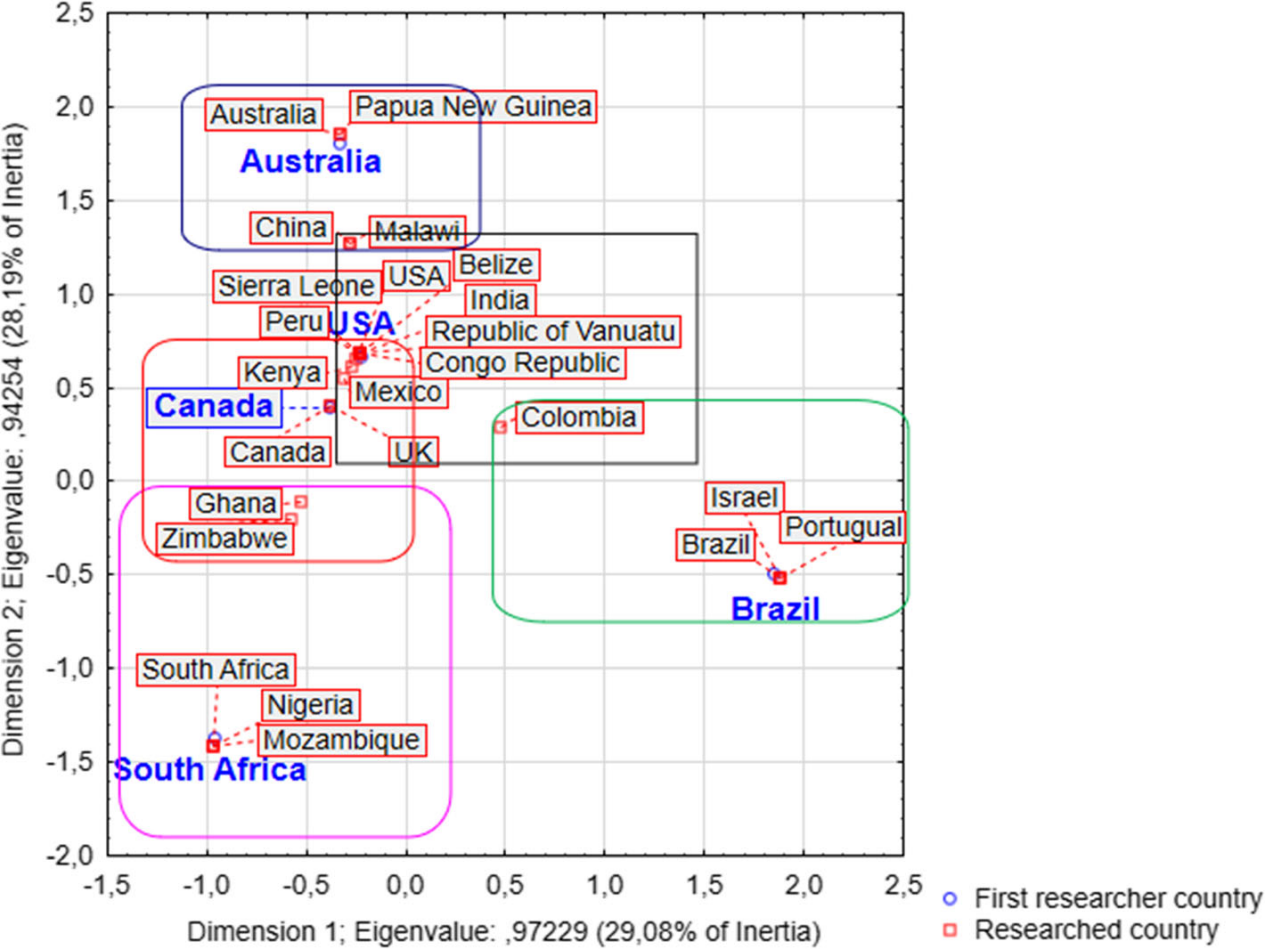
Correspondence analysis of the countries of origin of the first authors with the countries where the studies were carried out. Source: Databases searches of *Web of Science*, *Scopus*, *Science Direct*, and *Scielo*

Correspondence analysis identified two dimensions, which explained 29% and 28% of the total variance of the data, respectively. The accumulation of total variance of the data in the first two dimensions (57%) indicates the adequacy of the correspondence analysis since it was able to reduce considerably the dimensionality of the data (see Fig. 2).

A strong association was observed between authors from North American countries and research conducted outside their territories: USA (12 different countries from four continents) and Canada (seven different countries from three continents) (see Fig. 2)

Among the search terms related to ethnosciences, those that were most often found in the recovered works were those that made explicit references to “knowledge,” such as “indigenous knowledge,” “traditional knowledge,” and “ecological knowledge.” The only exception was “biocultural knowledge,” which was not found in the present study. Those terms containing the suffix “-ology” were less frequent than those in which the word “knowledge” was preceded by an adjective. The terms “ethnoecology” and “ethnopedology” were not present in any of the recovered works (see Table 3).

**Table 3:** Frequency of works that established articulations between local and scientific knowledge in the school context, obtained through the combined searches of terms of ethnoscience and terms of education, carried out using four databases and the English language

Terms of ethnoscience	Terms of education																				
	Aboriginal education	Aboriginal school	Alternative conception	Conceptual profile	Contextualized education	Education and school	Epistemological pluralism	Indigenous education	Indigenous school	Multiculturalism	Pedagogical practice	Rural education	School	School knowledge	Science classes	Scientific education	Student	Teacher	Teaching learning	Urban education	Total
Biocultural evolution	*	*	*	*	*	*	*	*	*	*	*	*	*	*	*	*	2	4	*	*	6
Biocultural knowledge	*	*	*	*	*	*	*	*	*	*	*	*	*	*	*	*	*	*	*	*	*
Cultural transmission	*	*	*	*	2	20	*	2	*	*	*	*	13	*	*	*	1	5	*	*	43
Ecological knowledge	1	*	*	*	3	26	1	*	*	3	1	*	36	*	*	1	12	12	*	*	96
Ethnobiology	*	*	*	*	*	8	1	*	*	*	1	*	8	3	*	*	5	3	*	*	29
Ethnobotany	*	*	*	*	*	10	*	*	*	1	*	*	12	*	2	*	4	4	*	*	33
Ethnoecology	*	*	*	*	*	*	*	*	*	*	*	*	*	*	*	*	*	*	*	*	*
Ethnopedology	*	*	*	*	*	*	*	*	*	*	*	*	*	*	*	*	*	*	*	*	*
Ethnozoology	*	*	*	*	*	3	*	*	*	*	*	*	1	*	*	*	1	*	*	*	5
Indigenous knowledge	*	*	1	*	*	139	*	20	1	10	5	1	168	2	4	*	47	43	4	*	445
Local Knowledge	*	*	*	*	*	57	*	3	1	3	*	3	44	2	3	*	33	37	*	1	187
Traditional ecological knowledge	*	1	*	*	2	24	*	*	*	4	*	*	28	*	*	*	20	10	*	*	89
Traditional knowledge	1	*	1	1	1	53	1	4	5	1	1	*	59	2	2	2	37	27	2	1	201
Native knowledge	*	*	*	*	*	4	*	*	*	*	1	*	3	*	*	*	3	4	*	*	15
Total	2	1	2	1	8	344	3	29	7	22	9	4	372	9	11	3	165	149	6	2	1149

Source: database searches of *Web of Science*, *Scopus*, *Science Direct*, and *Scielo*

Among the terms related to the field of education, those referring to the school environment itself (“*school*”) and to the agents directly involved in the educational process (“*student*” and “*teacher*”) were the most frequently found in the recovered works, while terms that indicated lines of thought were less frequent (see Table 3).

### Characterization of the objectives of the works

The recovered works that establish articulations between local and scientific knowledge in the school context represented several areas of knowledge. By the very nature of the search, works were recovered in the field of ethnoscience (e.g., “*Are identities oral*? *understanding ethnobotanical knowledge after Irish independence* (*1937*-*1939*)” [29] and “*A comparison of traditional plant knowledge between students and herders in northern Kenya*” [30]); as well as education (e.g., “*Advancing educational diversity*: *antifragility*, *standardization*, *democracy*, *and a multitude of education options*” [31] and “*An education rooted in two worlds*: *The Karen of northern Thailand*” [32]); or both areas (e.g., “*A truth*-*based epistemological framework for supporting teachers in integrating indigenous knowledge into science teaching*” [33]).

Other works that did not represent ethnoscience or education were also recovered, including areas such as sociology, anthropology, health, geology, mathematics, linguistics, and architecture, for example [34–40], respectively.

The objectives of works in which articulations between the two areas of knowledge in the school context were established also varied. Studies were recovered that aimed at the transmission of local knowledge in the school environment [41, 42], or addressed the detrimental influence of formal education on the knowledge systems of traditional people [30, 43]. Some works also included methods and techniques for teaching-learning processes, such as the use of didactic materials and sequences [44, 45]; or dealt with teacher education [46]; or analyzed the implications of using or not local knowledge in formal education systems and curricula; or even addressed epistemological conceptions that emerge from relationships between local and scientific knowledge [47, 48].

### Inclusion of local knowledge in formal education systems

The inclusion of local knowledge in educational systems is presented in the works as positive [10], and there can even be considered a consensus regarding its importance in the valorization and recovery of the knowledge and experiences of students [49]. This is probably because students, as sociocultural subjects, have knowledge, culture, and projects as fruits of their experiences [16]. Communities in which students live may thus be places filled with inspiration, which, if properly approached, will make students critical agents of their own realities [39].

Some positive contributions to the teaching-learning process represented among the recovered works include (1) production of contextual teaching involving the perspective of Science, Technology, Society, and Environment [9]; (2) socially just education, which privileges the reasoning skills of students and encourages them to value their cultures [50, 51]; (3) teaching-learning process that is active [50, 52], questioning, and preparatory for decision making [52]; (4) expansion of learning horizons with student recognition of the information built by cultural groups outside the dominant culture, and the exploration of local issues [13, 37, 52–54], possibility of learning multiple forms of interpretation of problems and phenomena useful in several situations [39, 53]; (5) conservation and perpetuation of local knowledge [8, 30, 55]; (6) use of the environment as a formal learning tool [39, 55, 56]; (7) use of local knowledge as a pedagogical, instructional, and communicative instrument for the educator [14]; and (8) reinforcing the sensitivity of teachers and researchers to the specific sociocultural contexts of students [57].

Some of the works also highlighted the supremacy of scientific knowledge, sometimes termed as Western knowledge. As referring to the debate on this problem in the works under analysis, we identified the following main aspects:

1. Discussion about the claimed universality of scientific knowledge and the fact that local knowledge does not have the formal aspects of standard science [47, 54, 58, 59] and can be overpowered by the dominant culture [56, 60].
2. The possibility of students from culturally diverse backgrounds being forced to accept values and assumptions that do not fit to their realities, as well as being prevented from examining values, assumptions, and information present in other cultural perspectives [56, 58, 60].
3. Identification of educators as agents that can deal with articulations between types of knowledge. They can recognize culturally situated knowledge about biodiversity, language, and values locally related to natural resources [8, 55, 61, 62].

Some works also report that teachers’ use of strategies that integrate knowledge can make all students in the classroom feel they are considered [13, 63], as well as strengthen family-school partnerships [63].

Other works also recorded the pertinence of the applicability of local knowledge in school curricula [11] making it a potential key tool for revitalizing biocultural diversity and enhancing the fulfillment of educational objectives [48, 64].

#### Theoretical bases of the works

The introductory texts of the recovered works presented the theoretical assumptions that guided them, but not all were explicit in this aspect. In some cases, works only provided information on the use of local knowledge in the school context (for example [8, 33, 37, 42, 65]), while others on the role of schools in the transmission of knowledge (e.g., [42, 66, 67]) or on schooling and loss of knowledge (e.g., [41, 68, 69]).

A total of seventy-two theoretical assumptions were found in the set of recovered works. These are listed in Table 4 with the sources cited by the respective authors.

**Table 4:** Theoretical assumptions presented by works that established articulations between local and scientific knowledge

Theoretical assumptions (*n* = 72)^a^	Concise definitions	Recovered works	Reference(s) cited by the recovered work(s)	N° of occurrences
Multiculturalism	Pedagogical movement in which the knowledge constructed by different types of cultures is treated fairly, with respect and recognition, and is taught in schools [70].	El-Hani and Bandeira (2008) [71] Horenczyk and Tatar (2002) [72] McKinley (2005) [11] Melo-Brito (2017) [73] Niculae (2014) [74] Pais (2011) [38] Vargas (2017) [75] Yore (2008) [57] Eijck and Roth (2007) [47]^1^ Quilaqueo and Torres (2013) [76]^2^ Gondwe and Longnecker (2015) [77]^3^	* Stanley and Brickhouse (1994)^1^ Quilaqueo and Quintriqueo (2008), Quilaqueo (2012)^2^ Aikenhead (1996), Aikenhead and Jegede (1999)^3^	11
Vygotsky’s theory of learning	Epistemology focused on the social construction of knowledge through interactive teaching and learning activities in the classroom [78].	Chang, Lee and Yen (2010) [53] Dopico and Garcia-Vazquez (2011) [79] Mutekwe (2014) [80] Mutekwe (2017) [78] Nashon and Madera (2013) [81] Sousa, Carvalho and Kambeba (2017) [44] Govender (2011) [82]^1^ Owusu-mensah and Baffour (2015) [83]^1,2^	* Vygotsky (1978)^1^ Derry (1999)^2^	08
Postcolonial theory (PCT)	Area of cultural and critical theory that addresses the way in which the works written by colonizers distort the experience and reality of the colonized. This approach also shows the presence and identity of the colonized, claiming their lost or distorted past [84].	El-Hani and Bandeira (2008) [71] Glasson et al. (2010) [85]^1^ Gonye and Moyo (2015) [86]^2^ Mukhopadhyay (2015) [87]^3^ Nashon and Madera (2013) [81]^4^ Ninnes (2000) [88]^5^	* Mapara (2009)^4^ Asante (1991)^2^ Goodley and Runswick-Cole (2010), Nelson and Prilleltensky (2005), Shakespeare (2013), Slee (2011)^3^ Gandhi (1998)^5^ Carter (2007), McKinley (2007)^1^	06
Critical pedagogy	Educational movement based on an education that trains students with awareness of freedom and the ability to recognize authoritarian tendencies. This approach seeks to emphasize the connection between knowledge and power [89].	Madusise and Mwakapenda (2014) [90] Snively and Corsiglia (1997) [52] Harris and Barter (2015) [91]^1^ Rincón and Olarte (2016) [39]^2^ Writer (2002) [92]^3^	* Giroux (2010)^1^ Freire (2000)^2^ Freire (1992)^3^	05
Constructivist approach	Pedagogical perspective that considers the construction of knowledge as a process based on the learners’ previous ideas and which is organized based on their interactions with information available in the environment [9].	Bejarano et al. (2014) [9] Raina (2011) [93] Stears, Malcolm and Kowlas (2003) [94] Vhurumuku and Mokeleche (2009) [95]	*	04
Toulmin’s (1958) Argumentation Pattern (TAP)	Interdisciplinary study model that illustrates the characteristics of an argument based on claims, data, guarantees, supports and refutations [96].	Hewson and Ogunniyi (2011) [97] Ogunniyi (2007a) [98] Ogunniyi (2007b) [99] Ogunniyi (2011) [100]	Toulmin (1958)	04
Cultural responsive pedagogy	Student centered teaching-learning process and its cultural context. The knowledge they bring to school is used to achieve better results [101].	Babbitt et al. (2015) [102] Coles-Ritchie, Monson and Moses (2015) [63]^1,2^ Rioux, Ewing and Cooper (2017) [103]^5^	* Gay (2000)^1^ Ladson- Billings (1995)^2^ Barnhardt and Kawagley (2008)^5^	03
Culturally responsive education	Theoretical approach according to which a student’s learning process is influenced by their culture, context and everyday experiences [10].	Augare et al. (2017) [104] Mack (2012) [10] Marker (2006) [105]	*	03
Epistemological pluralism	Philosophical position that recognizes that there are other knowledge systems besides science, each having greater relevance over the others within its own system [6].	Baptista (2010) [5] Melo-Brito (2017) [73] Taylor and Cameron (2016) [106]	*	03
Third space	Socially constructed, hybrid cultural spaces within which discourses and epistemologies can be articulated and deliberated through dialogue [107].	Buendía et al (2004) [108]^1^ Stevenson (2015) [109]^1^ Glasson et al. (2010) [85]^1^	Bhabha (1994)^1^	03
Interculturalism	Model for the integration and management of ethnocultural diversity [110]	Melo-Brito (2017) [73] Niculae (2014) [74] Webb and Radcliffe (2016) [111]	*	03
Culturally relevant pedagogy	Theoretical model that seeks to encourage acceptance and affirmation of students’ cultural identity while developing critical perspectives [112].	Mavuru and Ramnarain (2017) [113]^1^ Peña Sandoval (2016) [114]^2^	Weiland (2015)^1^ Paris (2012)^2^	02
Culturally responsive teaching (CRT)	Teaching-learning process that seeks greater efficiency in the education of ethnically diverse students using their cultural characteristics, experiences and perspectives as channels [115].	Rahmawati et al. (2017) [116]^1^ Rahmawati and Ridwan (2017) [117]^1^	Gay (2000)^1^	02
Community-based pedagogies	Curricula and practices that reflect the knowledge and understanding of the communities in which schools are located and where students and their families live [118].	Sharkey, Olarte and Ramírez (2016) [64]^1^ Rincón and Olarte (2016) [39]^2^	Sharkey and Clavijo Olarte (2012a)^1^ Freire (2000), Clavijo (2015a), Medina, Ramírez and Clavijo (2015), Rincón (2014), Reyes (2012), Sharkey (2012)^2^	02
Cultural Border Crossing	Learning process in which students start from the subcultures of their everyday worlds and move to the science subculture [119].	Aikenhead (1997) [61] Borgerding (2017) [120]	Aikenhead (1996)	02
Culture-based education	Approach that aims to build and enhance students’ linguistic, cultural, cognitive and affective strengths [121].	Yazzie-Mintz (2011) [122] Kana’iapuni et al. (2017) [121]^1^	* Demmert and Towner (2003)^1^.	02
Funds of knowledge	Approach based on the premise that people have culturally and historically accumulated knowledge in a body of knowledge and skills essential for their survival and well-being [123].	Ewing (2014) [124]^1^ Rincón and Olarte (2016) [39]^2^	Moll (1992)^1^ Murrell (2001)^2^	02
Pluralism	Perspective that incorporates alternative forms of knowledge, supports local cultural and ecological preservation and values diversity [59].	McKinley (2005) [11] Avery and Hains (2017) [59]^1^	* Kassan (2010)^1^	02

The superscript numbers from the second column match the superscripts from the third column

^a^Only theoretical assumptions that were in more than one work with articulations between local and scientific knowledge in the school context were presented

Source: Database searches of *Web of Science*, *Scopus*, *Science Direct* and *Scielo*

### Methodological contributions

We divided the techniques into two phases. Phase I techniques are those used to articulate local and scientific knowledge. Phase II techniques are those used to collect and/or record the impressions and effects of phase I results.

The technique most used in phase I was teacher training (*n* = 19). The next most frequent techniques were guided or field visits with students within communities (*n* = 11) and lessons/conversations/group interviews involving local experts (*n* = 10) (see Table 5).

**Table 5:** Phase I techniques carried in activities that establish articulations between local and scientific knowledge

#	Methodological techniques	Works	N° of occurrences
1	Teacher training	Armour et al. (2016) [125] Baptista (2015) [126] Baptista and Carvalho (2015) [127] Beer (2016) [128] Belay et al. (2005) [129] Chinn et al. (2014) [130] Govender (2011) [82] Johnson et al. (2014) [131] Mclaughlin and Whatman (2015) [132] Menezes et al. (2015) [133] Mhakure and Mushaikwa (2014) [134] Moss (2008) [135] Ogunniyi (2007a) [98] Ogunniyi (2007b) [99] Ogunniyi (2011) [100] Parmin et al. (2016) [136] Stevenson (2015) [109] Veintie (2013) [137] Verrangia and Silva (2010) [138]	19
2	Guided/field visit with students	Bandeira and Morey (2010) [139] Bang and Marin (2015) [140] Carrin (2015) [141] Dopico and Garcia-Vazquez (2011) [79] Glasson et al. (2010) [85] Harris and Barter (2015) [91] Johnson et al. (2014) [131] Keane (2015) [142] Pardo et al. (2015) [143] Valderrama-Pérez et al. (2015) [45] Jagger (2016) [60]	11
3	Lessons/conversations/community interviews with local experts	Aikenhead and Elliott (2010) [144] Baquete et al. (2016) [145] Guido et al. (2013) [146] Odochao et al. (2006) [32] Pardo et al. (2015) [143] Rioux et al. (2017) [103] Roa (2015) [147] Ruddell et al. (2016) [148] Singh (2010) [149] Valderrama-Pérez et al. (2015) [45]	10
4	Student interviews with experts, parents and grandparents	Bandeira and Morey (2010) [139] Chambers and Radbourne (2015) [150] Dopico and Garcia-Vazquez (2011) [79] Esa and Jiwa (2015) [151] Harris and Barter (2015) [91] Madiba and Mphahlele (2003) [152] Roa (2015) [147] Singh and Singh (2013) [8] Sousa et al. (2017) [44]	09
5	Gymkhana/game/contest/science fair	Anohah and Suhonen (2016) [153] Dublin et al. (2014) [37] Magnussen and Elming (2017) [154] Nkopodi and Mosimege (2009) [155] Owusu-mensah and Baffour (2015) [83] Pardo et al. (2015) [143] Singh (2010) [149] Singh and Singh (2013) [8]	09
6	Didactic sequence applied by teachers	Armour et al. (2016) [125] Lee et al. (2012) [156] Matang and Owens (2014) [157] Naidoo and Vithal (2014) [158] Rahmawati and Ridwan (2017) [117] Rahmawati et al. (2017) [116] Valderrama-Pérez et al. (2015) [45]	07
7	Student workshops	Anohah and Suhonen (2016) [153] Arenas and Cairo (2009) [159] Gomes (2014) [160] Keane (2015) [142] Pardo et al. (2015) [143] Sousa et al. (2017) [44]	06
8	Production of didactic material with teachers	Aikenhead and Elliott (2010) [144] Johnson et al. (2014) [131] Letsekha et al.(2014) [161] Meyiwa et al. (2013) [162] Rubio (2016) [163] Scaramuzzi (2010) [42]	06
9	Application of didactic material	Baptista and El-Hani (2009) [7] Dolphen (2014) [164] Marques et al. (2017) [165] Rubio (2016) [163]	04
10	Classroom observations	Bang et al. (2013) [166] Geldenhuys (2009) [167] Linares (2017) [168] Rioux et al. (2017) [103]	04
11	Photographs taken by students	Coles-Ritchie, Monson and Moses (2015) [63] Keane (2015) [142] Roa (2015) [147]	03
12	Songs	Croft (2002) [169] Dolphen (2014) [164] Pardo et al. (2015) [143]	03
13	Use of the Internet or social media by students	Rincón and Olarte (2016) [39] Sousa et al. (2017) [44] Harris and Barter (2015) [91]	03
14	Dance	Madusise and Mwakapenda (2014) [90] Pardo et al. (2015) [143]	02
15	Experiments	Grasser et al. (2016) [170] Pardo et al. (2015) [143]	02
16	Parent/expert interviews by teachers	Wood and Mcateer (2017) [171] Glasson et al. (2010) [85]	02
17	Student narratives	Coles-Ritchie, Monson and Moses (2015) [63] Bandeira and Morey (2010) [139]	02
18	Student tests	Dupuis and Abrams (2017) [172] Matang and Owens (2014) [157]	02
19	Text analysis	Ogunniyi (2000) [173] Ogunniyi (2011) [100]	02
20	Text production by students	Sousa et al. (2017) [44] Keane (2015) [142]	02
21	Video production by students	Grasser et al (2016) [170] Rovera (2017) [174]	02
22	Community participation in school administration and management	Duku and Salami (2017) [175]	01
23	Creation of didactic garden by students	Esa and Jiwa (2015) [151]	01
24	Develop a double-entry table (local/scientific knowledge)	Julio and Velarde (2016) [51]	01
25	Develop cartoon drawing with students	Essé et al. (2017) [36]	01
26	Development of a cyberatlas with the community	Caquard et al. (2009) [176]	01
27	Development of an atlas with students and the community	Taylor et al. (2014) [3]	01
28	Development of software to explore the mathematical aspects of local symbols	Babbitt et al. (2015) [102]	01
29	Development of specific curriculum, with teachers and school community	Kraipeerapun and Thongthew (2007) [177]	01
30	Free list made by students	Arenas and Cairo (2009) [159]	01
31	Glossary building by students	Madiba and Mphahlele (2003) [152]	01
32	Group discussion	Msimanga and Lelliott (2014) [178]	01
33	Interview with teachers	Dorner and Gorman (2011) [179]	01
34	Local tradition lessons with expert	Klein (2011) [180]	01
35	Making drawings for students	Babaian and Twigg (2011) [181]	01
36	Movie exhibition	Babaian and Twigg (2011) [181]	01
37	Native science field center	Augare et al. (2017) [104]	01
38	Personal meaning maps	Gondwe and Longnecker (2015) [77]	01
39	Platform usage	Maema et al. (2013) [182]	01
40	Practice in the community by making a sundial	Oliveira and Ferreira (2017) [16]	01
41	Project of raising chickens in the community	Keane et al. (2017) [183]	01
42	Seminar with students	Grasser et al. (2016) [170]	01
43	Student internships with local expert mentors	Carr et al. (2017) [65]	01
44	Training course with students and teachers	Ajayi (2014) [184]	01
45	Workshop using software with teachers and students	Eglash et al. (2006) [185]	01
46	Workshop with mothers	Ewing (2014) [124]	01

Source: Database searches of *Web of Science*, *Scopus*, *Science Direct*, and *Scielo*

As for phase II, 28 techniques were performed by the authors. Of the total number of works, 87 had carried out literature reviews or were theoretical essays. Another technique used was the interview, both collectively and individually, involving students and/or teachers (*n* = 59), relatives, community members, or local experts (*n* = 20); or focus groups/group discussions (*n* = 20). A high frequency was also recorded for questionnaires (*n* = 29), documentary analysis (*n* = 27), direct observation (*n* = 19), action research (*n* = 18), and ethnography (*n* = 17) (see Table 6).

**Table 6:** Phase II techniques performed in to collect the impressions and effects of the activities

#	Methodological techniques	Works	N° of occurrences
1	Review/Theoretical	Aikenhead (2017) [186] Aikenhead (1997) [61] Anazifa and Hadi (2017) [187] Arenas and Cairo (2009) [159] Asabere-Ameyaw et al. (2015) [188] Avanzi (2016) [189] Avery (2013) [190] Baptista (2010) [5] Baronnet (2017) [191] Bejarano et al. (2014) [9] Bhola (2002) [192] Bledsoe (1992) [193] Brown (2017) [194] Celani (2016) [195] Cobern and Loving (2001) [6] Coelho and Maurício (2016) [196] Conradie and Toit (2015) [197] Cordeur (2015) [198] Cost (2015) [199] Dussel (2009) [43] Eijck and Roth (2007) [200] El-Hani and Bandeira (2008) [71] Ferreira and Zitkoski (2017) [201] Fortunato (2017) [31] Garcia (2008) [66] Giardinetto (2003) [202] Gopinathan (2006) [203] Grange (2007) [204] Grauvogel (2015) [205] Hallinger (1998) [206] Harrington and Pavel (2013) [207] Heckenberg (2015) [208] Herrmann (2016) [40] Kawagley et al. (1998) [209] Kim (2017) [210] Kimmerer (2012) [211] Krugly-Smolska (1995) [212] Lowan-Trudeau (2017) [213] Maldonado-Alvarado (2016) [214] Marker (2006) [105] Martínez (2016) [215] Mccarter et al (2014) [216] Mckinley (2005) [11] Mckinley and Keegan (2008) [217] Meaney and Evans (2013) [218] Melo-Brito (2017) [73] Menefee and Asino (2014) [219] Meunier (2008) [220] Meunier (2010) [221] Meyer and Barker (1997) [222] Molina-Andrade and Mojica (2013) [223] Mueller and Tippins (2010) [200] Mutekwe (2014) [80] Mutekwe (2017) [78] Ng’asike (2014) [224] Nhalevilo (2012) [225] Niculae (2014) [74] Ogunniyi and Rollnick (2015) [226] Orozco (2015) [227] Pais (2011) [38] Peña Sandoval (2016) [114] Postiglione (2010) [228] Quilaqueo and Torres (2013) [76] Raina (2011) [93] Rapimán (2007) [229] Reis and Ng-A-Fook (2010) [230] Reyes-García (2013) [231] Rodríguez Gómez et al. (2016) [46] Roué (2006) [232] Rozzi (2012) [69] Saito (2014) [62] Sarangapani (2003) [233] Semali (1999) [234] Silva and Araújo (2015) [235] Snively and Corsiglia (1997) [52] Snively and Corsiglia (2001) [58] Sumida Huaman (2011) [236] Tippeconnic and Faircloth (2010) [237] Trommsdorff and Dasen (2001) [238] Trueba (2009) [239] Urrieta Jr (2015) [240] Vargas (2017) [75] Verrangia (2010) [241] Verrangia (2013) [242] Vhurumuku and Mokeleche (2009) [95] Wråkberg and Granqvist (2014) [243] Yore (2008) [57]	87
2	Interview with students and/or teachers	Ajayi (2014) [184] Anwari and Sulistyowati (2016) [55] Armour et al. (2016) [125] Arofah (2017) [244] Baptista (2015) [126] Baptista and Carvalho (2015) [127] Baptista and El-Hani (2009) [7] Bejarano et al. (2014) [9] Borgerding (2017) [120] Cardoso and Araújo (2012) [245] Carrin (2015) [141] Chang, Lee and Yen (2010) [53] Coles-Ritchie, Monson and Moses (2015) [63] Croft (2002) [169] Cruz-Casallas, Guantiva-Sabogal and Martínez-Vargas (2017) [68] Demps et al. (2015) [67] Dolphen (2014) [164] Dublin et al. (2014) [37] Esa and Jiwa (2014) [151] Fuhai (2017) [246] Geissler (1998) [247] Govender (2011) [82] Klein (2011) [180] Kovalski and Obara (2013) [49] Lee et al. (2012) [156] Ma (2011) [248] Mack et al. (2012) [10] Magnussen and Elming (2017) [154] Marques et al. (2017) [165] Matang and Owens (2014) [157] Mavuru and Ramnarain (2017) [113] McCarter and Gavin (2011) [48] Mukhopadhyay (2015) [87] Naidoo and Vithal (2014) [158] Nashon and Madera (2013) [81] Perrelli (2008) [249] Prout (1985) [250] Rahmawati and Ridwan (2017) [117] Rahmawati et al. (2017) [116] Rojas-Maturana and Peña-Cortés (2015) [251] Rubio (2016) [163] Ruiz-Mallén et al. (2009) [252] Sepulveda et al. (2015) [253] Shannon et al. (2017) [29] Sharkey, Olarte and Ramírez (2016) [64] Shizha (2008) [254] Shizha (2014) [255] Stears, Malcolm and Kowlas (2003) [94] Sugiono, Skourdoumbis and Gale (2017) [50] Thomas, Teel and Bruyere (2014) [256] Thomson (2003) [257] Veintie (2013) [137] Verrangia and Silva (2010) [138] Webb (2013) [258] Webb and Radcliffe (2016) [111] Wiener and Matsumoto (2014) [259] Wyndham (2010) [35] Yazzie-Mintz (2011) [122] Zinyeka, Onwu and Braun (2016) [33]	59
3	Case study	Aikenhead and Elliott (2010) [144] Anazifa and Hadi (2017) [187] Aravena (2017) [260] Bang and Marin (2015) [140] Bang et al. (2013) [166] Baptista and Carvalho (2015) [127] Berkley (2001) [261] Cameron et al. (2004) [262] Caquard et al. (2009) [176] Carr, Kenefic and Ranco (2017) [65] Chinn et al. (2014) [130] Dei (2002) [14] Dopico and Garcia-Vazquez (2011) [79] Dorner and Gorman (2011) [179] Eglash et al. (2006) [185] Ewing (2014) [124] Ferreira and Zitkoski (2017) [201] Goldenberg and Gallimore (1991) [263] Gondwe and Longnecker (2015) [77] Grauvogel (2015) [205] Heckenberg (2015) [208] Kawagley et al. (1998) [209] Keane et al. (2017) [183] Kim (2017) [210] Linares (2017) [168] Lowan-Trudeau (2017) [213] Madiba and Mphahlele (2003) [152] Maldonado-Alvarado (2016) [214] Matemba and Lilemba (2015) [264] Mclaughlin and Whatman (2015) [132] Meaney and Evans (2013) [218] Meunier (2008) [220] Morcom (2017) [265] Niculae (2014) [74] Odochao et al. (2006) [32] Ogunniyi (2007a) [99] Ogunniyi (2007b) [98] Ogunniyi (2011) [100] Owusu-Mensah and Baffour (2015) [83] Pardo et al. (2015) [143] Parmin et al. (2015) [136] Roué (2006) [232] Roué (2006) [232] Rovera (2017) [174] Ruddell et al. (2016) [148] Sarangapani (2003) [233] Shizha (2008) [254] Singh and Singh (2013) [8] Vhurumuku and Mokeleche (2009) [95]	49
4	Questionnaire	Anohah and Suhonen (2016) [153] Bandeira and Morey (2010) [139] Beer (2016) [128] Cardoso and Araújo (2012) [245] Coles-Ritchie, Monson and Moses (2015) [63] Croft (2002) [169] Essé et al. (2017) [36] Geissler (1998) [247] Gonye and Moyo (2015) [86] Grasser, Schunko and Vogl (2016) [170] Horenczyk and Tatar (2002) [72] Hwa and Kai-Lung (2016) [266] Kana’iapuni et al. (2017) [121] Kovalski, and Obara (2013) [49] Mhakure and Mushaikwa (2014) [134] Millán et al. (2017) [267] Nashon and Madera (2013) [81] Ogunniyi (2000) [173] Ogunniyi (2007a) [99] Ogunniyi (2007b) [98] Pauka, Treagust and Waldrip (2005) [268] Quintriqueo et al. (2011) [269] Ruiz-Mallén et al. (2009) [252] Seraphin (2014) [13] Singh and Singh (2013) [8] Taylor et al. (2014) [270] Vlaardingerbroek (1990) [271] Webb (2013) [258] Wood and McAteer (2017) [171]	29
5	Documentary analysis (books, exams, archives, research, didactic materials, curriculum, platform)	Aravena (2017) [260] Arofah (2017) [244] Breidlid (2009) [272] Chu (2015) [273] Croft (2002) [169] Demps et al. (2015) [67] Dempster and Hugo (2006) [274] Dupuis and Abrams (2017) [172] Erduran and Msimanga (2014) [275] Fyhn (2014) [276] Glasson et al. (2010) [85] Gondwe and Longnecker (2015) [77] Keane (2008) [142] Klein (2011) [180] Ladio and Molares (2013) [41] Maema et al. (2013) [182] Matemba and Lilemba (2015) [264] Melo-Brito (2017) [73] Morcom (2017) [265] Mukhopadhyay (2015) [87] Ninnes (2000) [88] Reyes-García et al. (2010) [12] Scaramuzzi (2010) [42] Shannon et al. (2017) [29] Sugiono, Skourdoumbis and Gale (2017) [50] Taylor and Cameron (2016) [106] Veintie (2013) [137]	27
6	Focal group interview/group discussion	Arofah (2017) [244] Buendía et al (2004) [108] Chinsembu et al. (2011) [277] Duku and Salami (2017) [175] Essé et al. (2017) [36] Gonye and Moyo (2015) [86] Govender (2011) [82] Hewson and Ogunniyi (2011) [97] Jagger (2016) [60] McCarter and Gavin (2014) [278] Mukhopadhyay (2015) [87] Parmin el al (2015) [54] Quigley et al. (2014) [56] Rubio (2016) [163] Singh (2010) [149] Singh-Pillay, Alant and Nwokocha (2017) [279] Stears, Malcolm and Kowlas (2003) [94] Veintie (2013) [137] Vlaardingerbroek (1990) [271] Yazzie-Mintz (2011) [122]	20
7	Interview with relatives or local or community experts	Avery and Hains (2017) [59] Chang, Lee and Yen (2010) [53] Demps et al. (2015) [67] Duku and Salami (2017) [175] Geissler (1998) [247] Kraipeerapun and Thongthew (2007) [177] Lee et al. (2012) [156] McCarter and Gavin (2011) [48] Nashon and Madera (2013) [81] Nuñez (2004) [280] Odochao, Nakashima and Vaddhanaphuti (2006) [32] Pauka, Treagust and Waldrip (2005) [268] Prout (1985) [250] Rojas-Maturana and Peña-Cortés (2015) [251] Rubio (2016) [163] Sepulveda et al. (2015) [253] Stears, Malcolm and Kowlas (2003) [94] Thomson (2003) [257] Webb (2013) [258] Zinyeka, Onwu and Braun (2016) [33]	20
8	Direct observation/observation/non-participatory observation	Ajayi (2014) [184] Anwari and Sulistyowati (2016) [55] Baptista and El-Hani (2009) [7] Bejarano et al. (2014) [9] Borgerding (2017) [120] Dolphen (2014) [164] Essé et al. (2017) [36] Fuhai (2017) [246] Msimanga and Lelliott (2014) [178] Naidoo and Vithal (2014) [158] Nashon and Madera (2013) [81] Rahmawati and Ridwan (2017) [117] Rahmawati et al. (2017) [116] Stears, Malcolm and Kowlas (2003) [94] Sugiono, Skourdoumbis and Gale (2017) [50] Valadares and Silveira-Júnior (2016) [281] Valderrama-Pérez, Andrade and El-Hani (2015) [45] Webb and Radcliffe (2016) [111] Yazzie-Mintz (2011) [122]	19
9	Action research	Apodaca (2013) [282] Augare et al. (2017) [104] Babbitt et al. (2015) [102] Chambers and Radbourne (2015) [150] Gomes (2014) [160] Guido et al. (2013) [146] Jiménez, Gullo and Montes (2016) [283] Johnson et al. (2014) [131] Keane (2008) [142] Krugly-Smolska (1995) [212] Letsekha et al. (2014) [161] Lewandowski (2012) [34] Marqui and Beltrame (2017) [284] Moss (2008) [135] Murillo (2009) [285] Ruiz-Mallén et al. (2009) [252] Sousa, Carvalho and Kambeba (2017) [44] Writer (2002) [92]	18
10	Ethnography/autoethnography	Apodaca (2013) [282] Berkley (2001) [261] Duku and Salami (2017) [175] Huaman and Valdiviezo (2014) [286] Jiménez, Gullo and Montes (2016) [283] Krugly-Smolska (1995) [212] Lee et al. (2012) [156] Lewandowski (2012) [34] Madusise and Mwakapenda (2014) [90] Menezes et al. (2015) [133] Murillo (2009) [285] Reta (2010) [287] Rodrigues Marqui e Beltrame (2017) [284] Rubio (2016) [163] Ruiz-Mallén et al. (2009) [252] Sousa, Carvalho and Kambeba (2017) [44] Veintie (2013) [137]	17
11	Participant observation	Bandeira and Morey (2010) [139] Belay, Edwards and Gebeyehu (2005) [129] Berkley (2001) [261] Ewing (2014) [124] Grasser, Schunko and Vogl (2016) [170] Harris and Barter (2015) [91] Kovalski and Obara (2013) [49] Madusise and Mwakapenda (2014) [90] Nkopodi and Mosimege (2009) [155] Nuñez (2004) [280] Rubio (2016) [163] Shizha (2008) [254] Singh and Singh (2013) [8] Thomas, Teel and Bruyere (2014) [256]	14
12	Analysis of drawings/mental maps/phrases/text	Bastos et al. (2016) [288] Carrin (2015) [141] Oliveira and Ferreira (2017) [16] Parmin et al. (2015) [54] Parmin et al. (2016) [136]	5
13	Discourse analysis	Handa and Tippins (2013) [289] Valderrama-Pérez, Andrade and El-Hani (2015) [45]	2
14	Guided visit	Bejarano et al. (2014) [9] Bruyere, Trimarco and Lemungesi (2016) [30]	2
15	Not applicable	Babaian and Twigg (2011) [181] Beer and Wyk (2011) [290]	2
16	Photograph analysis	Roa (2015) [147] Thomas, Teel and Bruyere (2014) [256]	2
17	Reflective journals	Rahmawati et al. (2017) [116] Rahmawati and Ridwan (2017) [117]	2
18	Revalorized participatory research	Núñez (2008) [291] Vargas (2017) [75]	2
19	Analysis based on narrative inquiry	Baquete et al. (2016) [145]	1
20	Comparative board	Pardo et al. (2015) [143]	1
21	Focal monitoring	Boyette and Hewlett (2017) [292]	1
22	Free list	Ladio and Molares (2013) [41]	1
23	Interviews by vignettes	Quigley et al. (2014) [56]	1
24	Interviews with school administrators	Buendía et al. (2004) [108]	1
25	Record of photographs by interviewees	Quigley et al. (2014) [56]	1
26	Video analysis	Magnussen and Elming (2017) [154]	1
27	Video observation	Magnussen and Elming (2017) [154]	1
28	Workshop	Ladio and Molares (2013) [41]	1

Source: Database searches of *Web of Science*, *Scopus*, *Science Direct*, and *Scielo*

## Discussion

The last decade has seen a quantitative increase in research works connecting different sources of knowledge in the school environment. Nevertheless, the data presented here reveal that the emergence of this kind of research is very recent (i.e., 1985). Furthermore, the number of works per year was very low in the two decades following the first recovered publication (Fig. 1).

Considering that the first formal specific publications in the ethnosciences arose at the end of the nineteenth century [293], a relatively long time elapsed (nearly one century) until the publication of the first works connecting ethnosciences and education. This gap seems to be related to the lack of connection between ethnoscientific published works on the one hand, and western studies on pedagogy on the other hand. A comment on this gap was made by French philosopher Bruno Latour (1987) [294] who noticed that studies on ethnoscience were “far remote from pedagogy.” Shortly thereafter, in the early 1990s, Brazilian educator Paulo Freire [295] registered a growth in ethnoscientific studies in Brazil and raised the possibility of relating those studies with the teaching-learning process. This means that, although a gap was noticeable, new research was being done to address that problem.

In “Pedagogy of the oppressed,” one of his seminal works, Freire [296] considered that education may be a domination practice that often reinforces the naïveté of students and their accommodation to serve the dominating class. This way of thinking is endorsed by the fact that the so-called local knowledge in the works recovered here generally arises from life experiences of culturally oppressed people. Thus, the late emergence of these recovered works, as well as the relatively slow growth of the “hybrid” research field that they represent, may be interpreted as a historical consequence of dominance over the last centuries.

Thus, through political actions, many local people have achieved some political awareness of the relevance of the local knowledge they bear. This seems to be related to what Hunn [2007] calls the fourth phase in the history of ethnobiology, in which local people can consciously judge and influence the objectives and methods of research and education works involving their participation.

This political awareness can sometimes result in the fruitful inclusion of local knowledge in formal teaching-learning experiences in various cultural contexts. Relevant examples come from multicultural education practices in the USA, Canada, and Australia, where cultural minorities have been submitted to strong oppression [47].

Regarding this possibility of sociocultural inclusion, the report of the International Commission on Education to UNESCO on 21st century education (1996) questioned the standardization of education as a result of globalization and the consequent damage to minority cultures. This report challenges the new century by proposing an education that will awaken a democratic civic culture while at the same time stimulates mutual respect of cultures based on the collective rights of all peoples on the planet [297]. The results of the present study show that, only 12 years later, the field of research under investigation exhibited increasing results in relation to the connection of knowledge proposed by UNESCO.

Almost all of the ten journals of the present study with more than three recovered works are included in the *Scimago Journal & Country Rank* [27], with the exception being “*Ciência & Educação*.” This latter journal is ranked in Plataforma Sucupira [298], which confirms its authenticity. Only one of these ten journals was in the field of ethnoscience (Table 2). This finding calls attention to the need for greater involvement of ethnoscientists in education.

All of the main countries of origin of the first authors of the recovered works (USA, Brazil, Canada, Australia, and South Africa) were colonized by European countries (Fig. 2). Furthermore, in all of them, indigenous people resisted cultural marginalization and homogenization. Attempts to integrate indigenous cultural heritage in formal curricula also occurred in all of these countries [260].

As for works specifically related to the ethnosciences, the relatively high frequency of works that used expressions such as “adjective + knowledge,” as compared to those using terms with the prefix “ethno-” (Table 3), is in agreement with the results obtained by the Brazilian ethnobiologist Natalia Hanazaki [143]. This author found more journals that published themes related to *traditional ecological knowledge* than those that used the “ethno-” prefix, with the use of this prefix being more prominent among Brazilian researchers.

The absence of results from the field of ethnoecology (Table 3) does not reflect the scenario found by a study that aimed to analyze the set of ethnoecological research until 2012, showing that the respective number of publications was growing despite ups and downs [299]. The study also only used the terms “ethnoecology” and “ethnoecological” in its search methodology. Since this field is hybrid in nature (between the natural and social sciences), it is likely that all the publications in this area were not compiled, since there are investigations that contribute to the field yet do not use these terms [299]. This same phenomenon may have occurred in the present study. It may also indicate that despite the great potential for connection between ethnoecology and education, there is a gap to be explored in this field, or that studies with ethnoecological characteristics are being incorporated by other fields, such as ethnobiology and studies on traditional ecological knowledge.

The variety of areas of research represented by the works recovered using the combination of terms from the fields of education and ethnoscience shows a potential to be harnessed, with contributions from different perspectives. This means that knowledge connections may, in principle, encompass all the disciplines that compose school curricula, thus enabling a comprehensive student formation, especially if applied in an interdisciplinary way (see [300] and [301]).

In general, judging from the content of the works recovered here, their main objective was not to take local knowledge as a substitution for science. This view reinforces the possibility for conceptual profile change (when a student keeps their previous knowledge and combines it with science) as presented by Mortimer [302].

Another common trend found here was that of the coexistence of different kinds of knowledge within teaching systems. This seems to be in agreement with the ideas of some authors related to argumentation theory [98, 302] and to epistemological pluralism [17, 99].

Multiculturalism, Vygotskian theory of learning, postcolonial theory, constructivism, critical pedagogy, and the argumentation theory were the main theoretical bases of half of the recovered works in which some explicit theoretical orientation could be found (Table 4). Some aspects are common to all of these theories: an active attitude of students in the teaching-learning process, the role of the teacher as someone who will mediate and stimulate this process, and the development of a critical attitude in relation to the dominant culture.

Regarding the relationship between culture and scientific education, emphasis on the critical attitude may be related to factors such as the growth of constructivism, the increase in the number of studies on the historical processes of curriculum development, growing critical attitudes of social groups in the face of western science, and the fact that western science often does not recognize other kinds of knowledge [17].

The fact that most of the recovered works are not based on, or do not clearly present, a guiding theoretical framework, suggests an opportunity for researchers to explicitly ground their studies in some theoretical framework.

The diversity of theoretical assumptions presented by the recovered works indicates a potential for connecting different types of knowledge, while deepening and valuing each (Table 4). A researcher can therefore find contributions that amplify the beneficial effects of this association. The same can be said for the diversity of methodological techniques applied to the study or establishment of connectivity between types of knowledge in the school context.

Despite the diversity of theoretical assumptions and techniques of the data collected (Tables 5 and 6), a study that analyzed the inclusion of local knowledge in the formal school system affirmed that education reformers, ethnobiologists, and cultural conservation professionals request this connection between different types of knowledge, but that few studies have examined this more detailed approach to the subject [48]. It is possible that the data presented by the present research will serve as an initial basis for future work from this perspective. It is noteworthy that it was not always easy to identify the methodological approach used in each work, which can make it difficult to replicate or analyze such research.

Some aspects of the present findings indicate that studies connecting scientific and local knowledge in schools represent a relatively new research field. These include the recent growth in the number of works per year, the concentration of most researchers in a small number of countries (Fig. 2), the small number of works for each author, and the high diversity of theoretical and methodological approaches (Tables 5 and 6). However, being “young” and expanding are not necessarily negative features of a field of inquiry. In fact, the diversity found here seems to indicate that different worldviews may be respected and valued, not only in the basic school context but also in the relationships among academic scientists who study the connections of local and scientific knowledge in the school context. We must finally stress the political necessity of reinforcing this connection in a permanent way.

## Conclusions

The diversity of recovered works demonstrates the interdisciplinary nature of the knowledge fields we studied. Thus, ethnoscientists willing to search for connections with education may find valuable information in such diverse labels such as health, mathematics, geography, and especially education sciences.

Observing the results applied in various sociocultural realities reveals that the viability of this articulation in the teaching-learning process is widely effective. It seems to be effective not only in the educational context of communities classified as traditional, but in many other education-related scenarios, involving many types of students, be they indigenous, aboriginal, children of fishermen, farmers, artisans, *quilombolas*, or from urban environments. Since most of our results were concentrated in South Africa, the USA, and Brazil, we reinforce the need for further studies of this kind in other parts of the world.

The lack of a clear indication of a theoretical basis in many of the works suggests a need for researchers interested in establishing this type of relationship do delve deeper into epistemological issues. On the other hand, the diversity of methodological approaches we found shows a promising scenario in terms of ways of bridging knowledge from different cultural sources in educational research.

Teachers are a fundamental component in this process of searching for an education that values knowledge diversity and establishes articulations. For this, they need initial (for new professionals) and continuous (already active professionals) training, besides time for further investigation on the contexts of students and greater incentive for the activities that promote the integration of knowledge.

Finally, it is necessary to take advantage of the school environment as a place of integration and recognition of the community through public policies and effective actions of articulation of scientific (based on curricula) and local (rooted in the communities around the school) knowledge. The community must be attracted to be part of these spaces, being welcomed in view of the great importance of the knowledge built and shared by its individuals over generations and that constitute the sociocultural framework of the subjects in formation that the school receives every day.

## Data Availability

Data sets analyzed as part of the current study are available from the corresponding author upon request.
